# Application of the socio-ecological model to understand the drivers of excessive alcohol and salt consumption: a qualitative study in Ghana

**DOI:** 10.1136/bmjopen-2025-100490

**Published:** 2025-10-23

**Authors:** Joseph Prince Mensah, Robert Akparibo, Chloe Thomas, Richmond Aryeetey, Alan Brennan

**Affiliations:** 1 School of Medicine and Population Health, The University of Sheffield, Sheffield, UK; 2 School of Public Health, University of Ghana, Accra, Ghana

**Keywords:** PUBLIC HEALTH, QUALITATIVE RESEARCH, NUTRITION & DIETETICS

## Abstract

**Abstract:**

**Objectives:**

This study aims to identify the key factors driving excessive alcohol and salt consumption in Ghana, both of which are modifiable risk factors for diseases such as cardiovascular conditions and cancers. Using the socio-ecological model (SEM), we qualitatively examine stakeholder perspectives to gain a comprehensive understanding of the influences contributing to these unhealthy consumption patterns.

**Design and methods:**

A qualitative study was conducted using semi-structured interviews. Transcripts were analysed thematically, with identified drivers mapped onto the corresponding levels of influence within the SEM.

**Participants:**

The study included 21 purposively sampled stakeholders from government and academic institutions in Ghana, including policymakers, practitioners and researchers.

**Results:**

Drivers of excessive salt and alcohol consumption were identified across all five levels of the SEM. At the intrapersonal level, disregard for health risks was a key factor. Community-level drivers included easy access to unhealthy foods and cultural norms promoting alcohol use at social events and salt in traditional dishes. At the societal and policy levels, inadequate regulation of the alcohol and food industries was found to reinforce lower-level drivers, further encouraging unhealthy consumption.

**Conclusions:**

This study highlights the multilevel influences on alcohol and salt consumption, emphasising the interactions across SEM levels. It highlights that addressing unhealthy consumption is not solely a matter of personal responsibility, demonstrating that societal and policy factors play a significant role in shaping health and dietary behaviours. The findings underscore the need for comprehensive public health strategies that address influences at multiple levels to effectively reduce excessive alcohol and salt intake.

**STRENGTHS AND LIMITATIONS OF THIS STUDY**

- A nutrition expert contributed to refining the interview guide, ensuring its relevance and comprehensiveness in capturing all potential drivers of excessive salt and alcohol consumption.
- Thematic analysis was conducted, with findings mapped onto the socio-ecological model, enabling the identification of meaningful themes aligned with the framework.
- Participants from the food and alcohol industries were under-represented, despite their significant influence on the supply and consumption of these products.
- The study did not explore potential interactions between salt and alcohol consumption at the individual level.
- Stakeholders’ views may not fully represent those of their institutions, even though participants were selected for their expertise.

## Introduction

 Harmful use of alcohol and excessive dietary salt consumption are well-established modifiable risk factors for non-communicable diseases (NCDs), including cardiovascular diseases, cancers and liver disease.^1 2^ High salt intake is a major contributor to hypertension, a key risk factor for stroke and heart disease, which are among the leading causes of global mortality.^3^ Similarly, harmful alcohol consumption is linked to cardiovascular diseases, liver cirrhosis, various cancers and mental health disorders.^4^ These risks are particularly pronounced in low- and middle-income countries (LMICs), where urbanisation and shifting dietary patterns have increased consumption, yet effective public health interventions remain limited.^5^ Importantly, excessive salt and alcohol consumption have synergistic effects, as both elevate blood pressure, strain cardiovascular and renal systems and exacerbate metabolic and liver dysfunction, thereby amplifying overall health risks.^3 6 7^

In Ghana, excessive salt and alcohol consumption represent growing public health concerns,^5^ given their synergistic effects on hypertension, cardiovascular disease, kidney dysfunction and liver disease. Although high consumption rates are well documented,^8^ key knowledge gaps remain regarding (1) the combined impact of salt and alcohol on disease risk in Ghanaian populations, (2) the sociocultural and economic determinants of excess consumption and (3) the effectiveness of integrated interventions that address both risk factors concurrently. Addressing these gaps is essential, as existing policies and programmes typically treat salt reduction and alcohol control in isolation. Evidence from this study provides important insights into the sociocultural and economic drivers of excessive salt and alcohol intake, while complementing our ongoing work aimed at tackling the other gaps identified.

The WHO recommends a daily salt intake limit of 5 g; however, the average intake in Ghana is 8.3 g per day, with 78% of the population exceeding the recommended limit.^9^ Both urban and rural populations show trends of high salt consumption which directly correlates with elevated systolic and diastolic blood pressure.^10^ In addition to the influence of globalisation and changing diets, the traditional high salt content of Ghanaian cuisine, for example, in soups, stews and preserved fish, also contributes significantly to excess intake.^5^ These trends mirror broader patterns in sub-Saharan Africa, where urbanisation exacerbates hypertension risk through increased salt intake.^11^

Alcohol consumption in Ghana is also deeply rooted in cultural practices, with variations in usage patterns and social purposes.^12^ However, patterns of use are increasingly hazardous. According to the WHO’s 2019 profile, alcohol use disorders in Ghana exceed the regional average, with 10% of adults engaging in binge drinking.^13 14^ The rapid expansion of the alcohol industry, coupled with evolving sociocultural and economic dynamics, is reshaping consumption behaviours and intensifying public health concerns.^15^ The combined burden of excessive salt and alcohol intake therefore represents an urgent and under-addressed public health challenge.

Ghana has implemented several policies to address excessive salt and alcohol consumption, including the National Alcohol Policy (2016) and the revised National NCD Policy (2022).^16 17^ These policies emphasise primary prevention through evidence-based and culturally relevant interventions.^18^ However, despite these efforts, challenges remain in addressing the underlying social, cultural and economic drivers of excessive consumption.^19^ A more comprehensive approach is needed, one that considers the complex interplay of broader personal, environmental and societal factors influencing alcohol and salt intake at multiple levels.^20 21^

The socio-ecological model (SEM) provides a valuable framework for understanding health behaviours by considering influences at multiple levels, including intrapersonal, interpersonal, community, societal and policy factors.^22^ Interpersonal-level and community-level factors encompass social relationships and norms, which can be influenced by the broader societal or political environment.^23^ While SEM has been widely applied to behaviours such as hazardous alcohol use,^24^ its application to salt consumption remains limited. Additionally, to our knowledge, no prior research has explored the shared drivers of alcohol and salt consumption in an African context using the SEM framework.

Addressing this gap, this study qualitatively examines stakeholder perspectives on the factors influencing unhealthy alcohol and salt consumption in Ghana. By mapping key drivers onto the SEM framework, this study provides a detailed analysis of the multilevel influences shaping these dietary and drinking behaviours. The findings offer critical insights for developing targeted, multifaceted public health interventions aimed at reducing the risks associated with excessive salt and alcohol intake.

## Methods

This study explored expert opinions on the drivers of excessive dietary salt intake and alcohol consumption in Ghana through qualitative interviews with key health and policy decision-makers. Interviews were employed as they allow for in-depth, open-ended discussions, enabling participants to elaborate on contextual and structural factors influencing these behaviours.^25^ Expert perspectives provide valuable insights into policy gaps, societal influences and intervention challenges that may not be fully captured through statistical data. The study followed the 32-item Consolidated Criteria for Reporting Qualitative Research checklist to ensure comprehensive reporting of qualitative research.^26^

### Sampling

Participants were recruited using maximum variation sampling and snowballing to capture a broad range of perspectives.^27^ The sampling frame included health policymakers, practitioners and researchers from key institutions in Ghana, identified as key informants due to their expertise and involvement in health policy-making or practice. A total of 25 stakeholders were initially approached, of whom 21 agreed to participate (table 1), with the remaining four declining due to scheduling conflicts. Recruitment was conducted via email, followed by phone calls to encourage participation.

**Table 1:** Participant profile

Participant groups in the study	Number
Public health agencies	n=6
Policymakers	n=2
Health practitioners	n=5
Health researchers	n=7
Civil society organisations	n=1

### Data collection

Data were collected using a semi-structured interview guide, developed based on the study objectives, a review of existing literature on salt and alcohol consumption drivers, and a situational analysis of Ghana’s public health landscape. The guide included open-ended questions on participants’ perceptions of alcohol and salt overconsumption, key influencing factors, potential mitigation strategies and government actions (see online supplemental material). It was refined with input from co-authors with nutrition and public health expertise and iteratively adjusted as new themes emerged during data collection. The guide also included questions on strategies to reduce overconsumption, which provided the basis for developing recommendations to address harmful alcohol and salt consumption.

All interviews were conducted in English, which is the official working language in Ghana. Interviews were conducted between October and December 2023, with 16 held face-to-face and 5 conducted virtually due to logistical constraints. Participants received an information sheet detailing the study’s purpose, potential risks and benefits, ensuring informed consent before participation. Interviews lasted 15-45 min and were audio-recorded with permission. Data collection was guided by the principle of data saturation, meaning interviews continued until no new themes emerged.

### Data analysis

Thematic analysis, supported by NVivo V.14,^28^ was used to systematically analyse interview data. The SEM provided a framework for structuring the analysis, categorising influences into five levels:^22^

1. Intrapersonal—individual knowledge, attitudes and behaviours.
2. Interpersonal—social relationships and peer influences.
3. Community—cultural norms and local environments.
4. Societal—broader societal and industry influence.
5. Policy—government regulations and policy implementation.

Analysis began with data familiarisation, where transcripts were reviewed to identify emerging patterns. Inductive coding was used to capture diverse ideas and perspectives without imposing preset themes. Initial codes were developed based on recurring concepts, emphasis placed by participants, and relevance to drivers of salt and alcohol consumption. Themes were iteratively mapped onto SEM levels, refined into sub-themes and continuously compared across cases to ensure consistency. To enhance validity, findings were peer-reviewed and refined through debriefing sessions. Additionally, initial results were presented to participants at a workshop in Ghana in May 2024, where feedback was incorporated before finalising conclusions. The purpose of the workshop was to validate and refine interpretation of the findings. Feedback largely confirmed the identified themes and provided minor clarifications, but no major discrepancies with the interview data were observed.

### Patient and public involvement

Patients and the general public were not involved in the design, analysis, reporting or dissemination of this study.

## Results

This study explored stakeholder perspectives on factors driving unhealthy alcohol and salt consumption in Ghana using the SEM. Findings reveal multilevel influences, spanning intrapersonal, interpersonal, community, societal and policy levels. Table 1 presents participant groups, and table 2 summarises the identified themes, illustrating how the drivers of excessive alcohol and salt consumption align with each level of the SEM, and the similarities between perceived drivers of consumption behaviours. Table 3 presents select stakeholder recommendations for interventions addressing these behaviours. The following sections detail specific themes for each SEM level, supported by stakeholder quotes illustrating insights into these unhealthy dietary behaviours.

**Table 2:** Themes for drivers of unhealthy consumption of alcohol and salt mapped onto the five levels of the socio-ecological model (SEM)

SEM levels	Main themes	Alcohol	Salt
Intrapersonal	Knowledge and attitude	Lack of knowledge about harm	Disregard for health risks
			Unawareness of healthy eating guidelines
	Perceptions and beliefs	Viewed as aphrodisiac	
		Perceived as antidepressant	
	Personal use and consumption behaviour	Addiction to alcohol	Prioritising taste
			Personal preference and habit
Interpersonal	Social circle influence	Peer pressure	Household cooking and food preparation behaviour
Community	Sociocultural factors	Normalisation of alcohol use during social and cultural events	Traditional dishes rich in salt
			Use of salt for food preservation
	Physical environment	Cheap and affordable alcohol	More ultra-processed/fast-food outlets
Societal	Social stressors	Unemployment	
	Media and industry influence	Alcohol media advertisements	Adverts of food high in salt
		Celebrity influence	
		Excessive promotion by industry	
Public policy	Regulation and monitoring	Inadequate industry and sales regulation	Inadequate regulation of food and salt-related industries
		Lack of penalties	
	Policy implementation		Lack of effective nutrition labelling policies
	Policy awareness	Lack of public awareness and support of alcohol control policies	Lack of awareness and adherence to policy
			Limited education on dietary guidelines

**Table 3:** Recommendations for preventing the overconsumption of alcohol and salt

Recommendations	Example quotes
Alcohol advertising restrictions: Implement restrictions on alcohol advertising to reduce consumption and promote public health.	“The usual advertisements should go down”—health researcher
Alcohol licence restrictions: Enforce alcohol licensing restrictions and regulations to limit availability and reduce consumption.	“Restricting the sale of alcohol to certain locations or specific places really will be very good”—health researcher
Alcohol taxation Implement increased or revised taxes on alcohol to discourage consumption.	“introduce some taxes that can discourage some consumption among at least some population”—health researcher “evidence has shown that if we increase taxes on these products like the sin taxes, then access or consumption tends to reduce. But for a very long time, Ghana hasn’t been able to increase taxes on these things until recently, early part of this year when there was a major breakthrough by stakeholders, civil society organisations, researchers and different stakeholders that push for that to happen.”—health practitioner “as for alcohol I think that we can impose some level of taxes on them. But if you look at our setting, a lot of the populace don’t take the expensive alcohol, it is the cheap alcohol our populace takes especially … it’s mostly the poor and the vulnerable who are taking some of the cheapest forms of alcohol we have in the country”—policymaker
Educational campaigns and health awareness campaigns: Provide educational programmes to promote healthier dietary choices and reduce alcohol intake.	“we need to educate people and gradually cause a behavioural change as to what they are supposed to or how they process those foods to reduce the amount of salt in there before consumption.”—health practitioner “I don’t think the education has come out very strongly. It’s something we can do more of because we all know salt is a major risk factor for hypertension too”—policymaker
Mandatory nutrition labelling: Require nutrition labelling to inform healthier food choices.	“we want to produce policies that will help, including mandatory nutrition labelling … we can get our front-of-pack labelling maybe policy and food marketing restriction policy, these two policies once we get these two policies out of that project, we can do so much”—policymaker
Mass media campaign Use mass media channels, such as TV, radio and social media, to promote healthier dietary behaviour.	“public campaign. So, that is where our gap is; we are not doing much. If you open your mass media, any of them, radio, TV, even social media, or the print media, you don’t see much as far as our campaign to healthy living, and all of these risk factors”—public health agency
Minimum age to purchase alcohol: Enforce a minimum age requirement for alcohol purchases to reduce underage drinking.	“you have to show something, there’s an identification to prove that indeed you are below 18 years but we don’t have that implemented yet. So probably it’s something that the government would want to look at”—public health agency
Sodium reformulation Work with local producers to implement sodium reformulation in food products to reduce overall sodium intake.	“companies or organisations that do processed foods and follow these guidelines, are taxed lower than companies who use more salt and all that. So yeah, I think it will be good.”—health researcher
Dietary guidelines: Develop and promote food-based dietary guidelines to encourage healthier eating habits.	“If you look at the dietary guidelines that was recently developed … it is something that is starting or emerging to support government, come out with these guidelines to make sure that we have these standardised measures in place to guide what we eat or to help us actually really understand what is good food and what is not good food.”—health practitioner

### Intrapersonal level

Key intrapersonal themes include (a) knowledge and attitude, (b) perceptions and beliefs, and (c) personal use and consumption behaviours. Stakeholders highlighted a widespread lack of awareness about the health risks of alcohol and salt consumption in Ghana. Many adults remain uninformed about the consequences of excessive drinking, with some struggling with addiction.

“[they] drink it as fun without knowing the consequences of what they are doing”—health researcher

Similarly, excessive salt consumption is driven by a lack of awareness or neglect of its health risks. Many Ghanaians are unaware of or disregard dietary guidelines, such as WHO’s recommendation of less than 5 g of salt daily.^29^

“[people] don’t really know the recommendation for salt intake, and our quantity a day, and people are just abusing it”—health practitioner

Taste often takes precedence over health considerations, as many prioritise flavour over the risks of high salt intake. Additionally, fatalistic attitudes, such as believing that dietary habits ultimately do not affect one’s fate, discourage behaviour change. This risky dietary behaviour may be driven by personal knowledge (or lack thereof) and could also be influenced by broader factors across other levels of the SEM.

“[people] like to take a lot of salt because of the taste it gives to food”—health researcher

“the perception and the mentality is that ‘Oh, we will all die, so whatever you do, you will die, so it doesn’t really matter what you eat’”—health researcher

Stakeholders also noted that alcohol is overconsumed for non-recreational purposes, including as an aphrodisiac or antidepressant. These culturally-rooted beliefs persist in Ghana despite limited evidence supporting such claims.^30 31^ This is further exacerbated by the promotion and advertising of these alcoholic concoctions with limited regulatory oversight.

“people are also advertising these alcoholic beverages for potency when it comes to men, a special potency. And so people are also jumping on that and drinking a lot of alcohol”—health practitioner

“People drown their sorrows in alcohol”—health researcher

Personal knowledge, beliefs and preferences are therefore significant drivers of excessive salt and alcohol consumption. These intrapersonal factors are further influenced by broader societal-level and policy-level forces within the SEM.

### Interpersonal level

At the interpersonal level, family relationships and peer interactions within social circles significantly influence both alcohol consumption and dietary salt intake, in either healthy or unhealthy ways. In particular, home cooking and food preparation habits play a major role in the excessive use of salt. It was noted that the liberal use of salt and seasoning often stems from household characteristics. Therefore, the issue may not only be due to a lack of knowledge or personal awareness about the health risks of excessive salt intake but also the influence of household norms that reinforce these unhealthy cooking practices or dietary behaviour.

“because we are not in homes of people, when they are cooking, they add salt. They add these spices too to the food. And it’s increasing the amount of the salt in the food”—public health agency

Stakeholders also noted that peer pressure within social circles is a key driver of harmful alcohol use. This is particularly evident in urban areas, where increased opportunities for social and corporate gatherings create environments where peers can influence each other to drink.

“peer pressure, outing with friends, corporate meetings, programmes that attract all these things… these are the behaviours that usually causes [harmful alcohol use]”—health practitioner

Peer pressure, as a driver of harmful use of alcohol, seems to interact with other sociocultural factors at the community level of the SEM in influencing or driving unhealthy behaviour, and therefore can only be fully understood in light of the wider interacting factors.

### Community level

Sociocultural influences and factors related to the physical environment were identified as community-level drivers of unhealthy consumption behaviours. Stakeholders reported that alcohol use has become normalised during cultural events and is now a common feature in homes and communities. This shift in attitudes towards alcohol consumption may be attributed not only to the urbanisation of communities, but also to other contextual factors within the society that may have made people more accepting of alcohol use.

“there are some occasions, traditional or more formal occasions, the use of alcohol for these occasions has become a norm”—health researcher

The cultural and social use of alcohol is closely tied to broader societal factors within the SEM, and if left unchecked, could have harmful effects on the population. In Ghana, the unhealthy consumption of salt is also influenced by cultural practices. In this context, the foods available for consumption tend to be high in salt, driving daily salt intake to exceed recommended levels. As one participant noted, traditional Ghanaian delicacies often contain high amounts of salt, further contributing to unhealthy consumption patterns.

“culturally, we are used to salty food, like ‘koobi’ and all those things”—health researcher

Additionally, sodium/salt has long been used culturally as a food preservative, particularly for meat and fish, due to its preserving properties.^32^ Stakeholders pointed out that the use of salt for food preservation is prevalent in the society and therefore this could pose a significant health problem since the consumption of foods may be accompanied with the excessive intake of salt.

“most of our [foods] are preserved in sodium”—health practitioner

Stakeholders also highlighted shifting consumption patterns as a key factor contributing to the unhealthy intake of alcohol and dietary salt. Dietary habits are changing, with more people consuming processed and fast foods. Furthermore, the increased presence of fast-food outlets in communities makes it easier to access unhealthy, salt-rich foods. This trend may be driven by rapid urbanisation in local communities across Ghana,^33^ leading to a rise in fast-food outlets and the availability of processed foods.

“we are beginning to see a rise in the use of ultra-processed foods, always very salted, they’ve got high salt content”—health practitioner

“apart from we preparing our own food in the house to eat, now people also buy a lot of fast foods and foods outside and all that”—public health agency

Additionally, the physical environment significantly contributes to the harmful use of alcohol. Stakeholders highlighted the greater opportunities for alcohol consumption, noting that it is cheap and readily accessible, which increases the likelihood of over-drinking.

“a lot of drinking spots have now opened. And in every nook and cranny, there are drinking spots and you’ll get people going to take alcohol”—public health agency

### Societal level

At the societal level, unemployment and other social stressors contribute to unhealthy alcohol consumption among the population. Participants suggested that the lack of employment can lead individuals to turn to alcohol as a coping mechanism, as the stress and depression associated with unemployment are understood to drive increased alcohol consumption.

“lack of employment, people idle, then for want of something better to do, they’ll be indulging in alcohol”—health researcher

Stakeholders also highlighted that the media and industry often undermine efforts to promote healthy eating and responsible alcohol consumption. They indicated that the media contributes to harmful alcohol use by excessively promoting alcohol beverages and featuring celebrity advertisements. The frequent portrayal of alcohol use in such adverts can entice the population to engage in drinking behaviours.

“most of the youth are into alcohol intake and of course sometimes, it is not their doing. It is the doing of the industry because of the excessive advertisement”—civil society organisation

### Public policy level

Another level identified in the SEM framework was public policy. There is generally a perception that monitoring and regulation of the alcohol, food and salt industries are inadequate. Stakeholders attributed this perception to weak enforcement of existing regulations and a lack of penalties or punitive measures aimed at controlling the sales and availability of alcohol and salt products in the market.

“promotion or marketing of these alcoholic beverages. It is everywhere, … and there’s no regulation”—health practitioner

Concerns were also raised about the government’s commitment to addressing these issues and the effectiveness of existing policies to control the problem. Stakeholders expressed that the government could play a more proactive role in promoting healthy lifestyle and dietary guidelines on a national level through its agencies and various channels. By increasing public awareness, support and adherence to these policies and guidelines, the government could significantly impact public health.

“It’s about awareness. It’s about monitoring, it’s about [government] actually making sure that people are adhering to the policy”—health practitioner

Furthermore, stakeholders reported challenges at the policy level regarding the implementation of food and salt-related policies. For example, the absence of effective nutrition labelling policies has resulted in an increased availability of processed foods high in salt in the market, ultimately leading to greater consumption of these products.

“we have been doing our best to make sure that labelling claims that are put on those products are tasteful and they make regulatory sense … But if somebody doesn’t make a claim, it goes meanwhile, the sodium content, the sugar content is very high”—policymaker

Despite the challenges associated with implementing alcohol and salt-related policies, stakeholders noted some recent successes in alcohol control measures. For example, stricter regulations have been introduced regarding celebrity advertising of alcohol, which is linked to influencing drinking behaviours at the societal level. Stakeholders acknowledged that successfully implementing alcohol and salt-related policies is not always straightforward, suggesting that barriers must be addressed for effective population health initiatives. However, the recent successes highlighted by stakeholders demonstrate that overcoming these challenges at the policy level is indeed possible.

“people copy the lifestyle of these people and so they have banned the use of celebrities in advertising alcohol”—health researcher

## Discussion

This study applies the SEM to identify factors influencing excessive salt and alcohol consumption, based on insights from key health and policy stakeholders. Findings reveal that these unhealthy dietary behaviours result from multiple influences across all SEM levels: intrapersonal, interpersonal, community, societal and public policy.^34^ This highlights the need for targeted interventions addressing drivers at each level to effectively mitigate the adverse health effects from overconsumption.

This study illustrates that excessive consumption of salt and alcohol is not solely a matter of individual choice; rather, it is shaped by broader interpersonal, societal and policy factors that warrant consideration in health interventions. Employing the SEM framework allows for a holistic understanding of health behaviours, acknowledging the interplay of individual, social, cultural and environmental factors.^34^ Evidence suggests that context-based interventions targeting environmental and societal influences often lead to more lasting impacts compared with approaches targeting only individual-level factors.^35^

The SEM suggests that health outcomes are influenced by interactions between individuals, communities and broader societal and political environments. To effectively reduce excessive salt and alcohol consumption, interventions must target all SEM levels. Sustained, multilevel efforts are crucial for fostering long-term behavioural changes. Health practitioners, researchers and policy makers can leverage the SEM to identify contributing factors and design comprehensive interventions targeting unhealthy consumption patterns.^23^

A significant driver identified was the lack of knowledge about the harms of unhealthy dietary behaviours, which is exacerbated by policy-level drivers such as limited education on dietary guidelines and poor adherence to alcohol control policies. The stakeholder-recommended interventions listed in table 3, such as educational campaigns or fiscal measures, could help address these gaps and encourage healthier dietary practices.^36^

One notable intervention is the Ghana Food and Drugs Authority’s ban on celebrity alcohol advertisements, initially implemented in 2015. This policy aimed to reduce alcohol consumption, especially among youth, by debilitating the influential role of public figures in promoting alcohol use.^37^ Such measures align with global efforts to limit alcohol marketing through influential personalities.^38^

At the societal and policy levels, inadequate regulation of the alcohol industry significantly influences community behaviours, thereby promoting harmful drinking patterns. A majority of Ghanaian adult alcohol consumers (57%) prefer locally produced beverages like *akpeteshie*,^39^ which often have high alcohol content, increasing health risks. Regulating production and access to these beverages is critical.^40^ While cultural norms contribute to alcohol consumption, research suggests that physical environments and the affordability of alcohol are stronger influences than cultural identity, especially among Ghanaians living abroad.^41^ Environmental interventions, such as licensing restrictions and taxation policies, could therefore reshape behaviours, promoting healthier consumption patterns.^42 43^

Stakeholder interviews provided critical, context-specific insights into the drivers of overconsumption. These interviews, conducted with experts knowledgeable about dietary and alcohol behaviours in Ghana, enrich the understanding of these issues. However, it is important to acknowledge that stakeholders’ views may not fully represent those of their institutions, even though participants were selected for their expertise. Additionally, the study would benefit from integrating insights from the food and alcohol industries. Including industry representatives could have added perspectives shaped by commercial interests, such as prioritising profit or promoting products, which may differ from the primarily public health-oriented views of policymakers, practitioners and researchers. Recognising these potential differences highlights the value of incorporating diverse voices in future research. To address this limitation, stakeholders from various sectors, including industry representatives, were invited to the validation workshop to confirm themes and ensure data saturation.

While this study identifies high-level influences on alcohol and salt consumption, it does not explore interactions between these dietary behaviours at the individual level. Further research should investigate these links, for example, whether alcohol use increases appetite for salty foods, or whether shared social and cultural settings reinforce concurrent consumption. Methodologically, mixed-methods or longitudinal approaches could provide more comprehensive insights into how alcohol and salt consumption influence one another over time.

Despite these limitations, the findings are broadly transferable to similar contexts. For example, studies in sub-Saharan Africa have also identified cultural norms, weak industry regulation and limited policy enforcement as key drivers of unhealthy consumption behaviours.^44 45^ Similarly, global literature highlights how alcohol marketing and the widespread availability of processed foods reinforce overconsumption across diverse settings.^46^ The identified drivers could therefore be examined in both LMICs and high-income countries to understand varying burdens and influences of unhealthy behaviours. This would allow for the development of tailored interventions suited to specific environments.

## Conclusion

Effective control of alcohol and salt consumption requires addressing the multilevel factors influencing their overuse. This study identifies multiple drivers across the five levels of the SEM, highlighting how interactions between these levels shape unhealthy dietary behaviours. It highlights that personal responsibility alone cannot account for these patterns; societal and political influences significantly affect dietary and drinking practices.

The study also highlights the importance of addressing these multilevel factors when designing public health strategies to mitigate the risks associated with excessive consumption. Stakeholder perspectives provide valuable insights into the drivers of salt and alcohol use, offering a comprehensive understanding that can inform the development of targeted interventions.

To combat the health risks of excessive alcohol and salt consumption effectively, public health and nutrition policies must address factors across all SEM levels. Examples include intrapersonal-level educational campaigns to raise awareness, community-based initiatives to shift social norms around food preparation and alcohol use, stronger industry regulation and licensing reforms at the societal level, and improved policy enforcement and nutrition labelling at the government level. Interventions that integrate intrapersonal-level, community-level, societal-level and policy-level measures are essential for achieving sustained behavioural changes and reducing the burden of non-communicable diseases in Ghana and similar contexts. Future research should examine how these multilevel interventions interact to produce lasting dietary changes in diverse cultural settings.

## Supplementary material

10.1136/bmjopen-2025-100490 online supplemental file 1

## Data Availability

Data are available upon reasonable request.
