# Supplementary material for: Application of the socio-ecological model to understand the drivers of excessive alcohol and salt consumption: a qualitative study in Ghana
Source: BMJ Open. 2025 Oct 23;15(10):e100490. doi: 10.1136/bmjopen-2025-100490 (PMC12551474; doi:10.1136/bmjopen-2025-100490)
Supplement: online supplemental file 1 [file bmjopen-15-10-s001.docx]

# Supplementary Material

## Supplementary Appendix 1:

Consolidated criteria for reporting qualitative studies (COREQ): 32-item checklist

Tong A, Sainsbury P, Craig J. Consolidated criteria for reporting qualitative research (COREQ): a 32-item checklist for interviews and focus groups. Int J Qual Health Care. 2007;19(6):349-357. doi:10.1093/intqhc/mzm042

| No. | Item | Comment | Reported on page number or not applicable (N/A) |
| --- | --- | --- | --- |
| **Domain 1: Research team and reflexivity** | | | |
| *Personal Characteristics* | |  |  |
| 1. | Interviewer/facilitator | The corresponding author (JPM) conducted the interviews | 4 |
| 2. | Credentials | MSc, MPH | N/A |
| 3. | Occupation | JPM was a PhD researcher at the University of Sheffield at the time of conducting the research | 1 |
| 4. | Gender | The interviewer was male | N/A |
| 5. | Experience and training | Interviewer has training in conducting qualitative health research, and all authors have extensive research experience | N/A |
| *Relationship with participants* | |  |  |
| 6. | Relationship established | The interviewer had no relationship with participants prior to recruitment into the study | 4 |
| 7. | Participant knowledge of the interviewer | The interviewer introduced himself to participants and explained the purpose of study, allowing for any questions, to ensure informed consent before participation | 4 |
| 8. | Interviewer characteristics | The interviewer was reported to be a researcher, with no bias concerning the research topic | N/A |
| **Domain 2: study design** | | | |
| *Theoretical framework* | |  |  |
| 9. | Methodological orientation and theory | Thematic analysis using the Socio-Ecological Model | 5 |
| *Participant selection* | |  |  |
| 10. | Sampling | Maximum variation sampling approach was used | 4 |
| 11. | Method of approach | Various methods of approach were used, including snowballing and email invitations to relevant institutions | 4 |
| 12. | Sample size | Twenty-one | 4 |
| 13. | Non-participation | Some key informants did not respond to their invitation to participate in the study. However, there were no drop outs among those who agreed to participate. | N/A |
| *Setting* | |  |  |
| 14. | Setting of data collection | Participants could decide whether to have the interview at their place of work or online | 5 |
| 15. | Presence of non-participants | None | N/A |
| 16. | Description of sample | Participant profile/description presented in Results section | 5 |
| *Data collection* | |  |  |
| 17. | Interview guide | The interview guide was pilot tested, and iteratively refined as interviews progressed | Supplementary file |
| 18. | Repeat interviews | No | N/A |
| 19. | Audio/visual recording | All interviews were audio-recorded and transcribed | 5 |
| 20. | Field notes | The interviewer wrote brief notes of any emerging themes during and after each interview | N/A |
| 21. | Duration | Interview times were generally between 15 to 45 minutes | 5 |
| 22. | Data saturation | Data saturation guided the conclusion of the data collection process | 5 |
| 23. | Transcripts returned | No | N/A |
| **Domain 3: analysis and findings** | | | |
| *Data analysis* | |  |  |
| 24. | Number of data coders | The interviewer and another transcriber coded the data, and the codes were discussed with all members of the research team regularly | 5 |
| 25. | Description of the coding tree | Coding was aided by the use of NVivo 14 software. | 5 |
| 26. | Derivation of themes | Themes were derived from the data | 5 |
| 27. | Software | NVivo was used to manage the data | 5 |
| 28. | Participant checking | Findings were presented to study participants for feedback | 5 |
| *Reporting* | |  |  |
| 29. | Quotations presented | Quotations were presented to illustrate the themes | 5-10 |
| 30. | Data and findings consistent | There was consistency between the data presented and the findings | 5-10 |
| 31. | Clarity of major themes | Major themes were clearly presented in the findings | Table 2 |
| 32. | Clarity of minor themes | Minor themes were clearly presented in the findings | Table 2 |

## Supplementary Appendix 2:

Interview guide

*Salt intake*

How important do you think excessive salt intake is in contributing to the risk of NCDs in Ghana?

- Have you observed a cultural shift around salt use in the population, and what could be the cause?
- Why do people use the amount of salt they use? What drives this unhealthy dietary behaviour?

*Alcohol consumption*

In the same way, how important do you think the harmful use of alcohol is in contributing to the risk of NCDs in Ghana?

- What is the scale of the problem in Ghana?
- What do you think are the key drivers, or contextual factors, influencing harmful use of alcohol in Ghana?

What consumption or alcohol purchasing behaviours are prominent in the population, and how do you think they could be controlled?

- What is the drinking behaviour?
- Are there ways in which strategies to reduce alcohol consumption in the population could be strengthened?

*Recommendations*

What standards have government set? Is there any guidance or national guidelines on safe use of alcohol / salt?

- What solutions are currently in place to solve this problem? Is there a recognised need to target the problem?
- How could these be put in place if non-existent?
- What are contextual/societal barriers of achieving salt and alcohol reduction targets?
- What are your recommendations, or what policies or interventions would you prioritise, that aims to target harmful use of alcohol and salt to prevent NCDs?

And why would you prioritise these policies?

- Do you know of any alternative measures to reduce the rising non-communicable disease burden (obesity/diabetes/cardiovascular disease etc.) in Ghana?
