# Supplementary material for: Application of the socio-ecological model to understand the drivers of excessive alcohol and salt consumption: a qualitative study in Ghana
Source: BMJ Open. 2025 Oct 23;15(10):e100490. doi: 10.1136/bmjopen-2025-100490 (PMC12551474; doi:10.1136/bmjopen-2025-100490)
Supplement: Supplementary file 2 [file bmjopen-2025-100490.reviewer_comments.pdf]

PEER REVIEW HISTORY

BMJ Open publishes all reviews undertaken for accepted manuscripts. Reviewers are asked to complete a checklist review form and are provided with free text boxes to elaborate on their assessment. These free text comments are reproduced below.

This paper was submitted to another journal from BMJ but declined for publication following peer review. The authors addressed the reviewers' comments and submitted the revised paper to BMJ Open. The paper was subsequently accepted for publication at BMJ Open.

ARTICLE DETAILS

Title (Provisional)

Application of the Socio-Ecological Model to understand the drivers of excessive alcohol and salt consumption: a qualitative study in Ghana

Authors

Mensah, Joseph Prince; Akparibo, Robert; Thomas, Chloe; Aryeetey, Richmond Nii Okai; Brennan, Alan

---

VERSION 1 - REVIEW

**Reviewer**	**1**
**Name**	**Reviewer 1**
**Affiliation**	
**Date**	**31-Jan-2025**
**COI**	

Comments not published pending reviewer's consent.

**Reviewer**	**2**
**Name**	**Reviewer 2**
**Affiliation**	
**Date**	**31-Jan-2025**
**COI**	

Comments not published pending reviewer's consent.

VERSION 2 - REVIEW

**Reviewer**	**3**
**Name**	**Jovicic-Bata, Jelena**
**Affiliation**	**University of Novi Sad, Department of Pharmacy**
**Date**	**07-Jul-2025**
**COI**	**None**

These are my comments to the respected authors.

Abstract

The abstract is informative enough and adequately summarizes the main findings.

Strengths and limitations

Well written.

Introduction

Introduction is comprehensively written and uses current references.

In the first paragraph, lines 37-45, it would be advisable to add at least some information on the usual salt content of traditional cuisine, as this is also an important determinant of the overall salt intake. The way this paragraph is written at the moment implies that globalization and urbanization are the sole drivers of excessive salt intake.

Materials and methods

This section is written concisely and is well organized, provides all the necessary elements.

Results

The results are easy to understand and follow, concise and comprehensive.

Page 6 if 20, lines 22-26, the Authors state that "Stakeholders also noted that alcohol is overconsumed for non-recreational purposes, including as an aphrodisiac or antidepressant. These beliefs persist despite limited evidence supporting such claims (27, 28)." Are these claims about alcohol being an aphrodisiac and/or antidepressant culturally based? If that is the case, that could be mentioned here, as in many other cultures, alcohol is not seen as such.

Discussion

Discussion is well-led, goes in line with the main findings and is comprehensive.

Page 10 of 20, lines 3-6: This might be the place to possibly elaborate in one paragraph on the underrepresentation of salt and alcohol industry representatives – What do the Authors think

would be the main differences between their answers compared to other stakeholders included in the study?

**Reviewer**	**4**
**Name**	**Thahir, Andi**
**Affiliation**	**The University of Sydney, Central Clinical School**
**Date**	**13-Jul-2025**
**COI**	**None**

Introduction

The flow of the introduction does not feel smooth, likely due to the way subheadings have been assigned to paragraphs. The authors should consider reorganising this section into three major areas: why salt and alcohol consumption matter in general, how prevalent and serious these issues are in Ghana, and what the current knowledge gaps are, along with potential solutions. Additionally, it remains unclear why salt and alcohol consumption in Ghana should be viewed as an urgent concern beyond the observation that both consumption rates are high. There is no discussion of whether these two factors exacerbate each other's health impacts, whether they share mechanistic pathways, or why their combined burden warrants particular attention. Clarifying these points would strengthen the rationale and make the introduction more compelling.

Method

Page 4, lines 43-45: You mentioned, *"The sampling frame included health policymakers, practitioners, and researchers from key institutions in Ghana. A total of 25 stakeholders were initially approached, of whom 21 agreed to participate (Table 1)."* Could the authors clarify what prevented the inclusion of all 25 participants? How did you determine when saturation of responses had been reached? Additionally, how was initial contact with participants made, and who were considered the key informants? Was the interview conducted in English or local languages such as Akan or Ewe?

Page 5, lines 23-25: It is stated that *"Additionally, initial results were presented to participants at a workshop in Ghana in May 2024, where feedback was incorporated before finalising conclusions."* Could you clarify what kind of feedback was received and what the purpose of collecting this feedback was? Did the feedback lead to changes in the initial results, particularly if there were discrepancies between the interview data and participant reflections during dissemination?

It would also be helpful to explain more specifically how initial codes were developed inductively. For example, what criteria guided the identification of patterns before mapping them onto the SEM levels?

I agree with the previous round of review asking, "*How was the interview guide developed, based on?*" Although the authors claimed that they had already revised it but the response and revision did not actually and sufficiently address it. They mentioned, "A nutrition expert contributed to refining the interview guide" How? Also, are these experts acknowledged in the acknowledgement or as co-authors?

Results

Table 2 shows that *"Delicacies rich in salt"* is categorised under the "*Sociocultural factors"* theme. However, this appears to reflect participants' opinions and perceptions, which are more likely shaped by their intrapersonal attitudes. Therefore, it may fit better under the "*Perceptions and Beliefs"* theme. Does this subtheme overlap with "*Prioritising taste"*?

Meanwhile, the "*Use of salt for food preservation"* seems more appropriately placed under the *Sociocultural factors* theme. What is your view on this?

Importantly, I think "*Household cooking and food preparation behaviour"* should be classified under the Community SEM level within the "*Sociocultural factors"* theme. If this reclassification is made, then the *Interpersonal* domain cannot be represented solely by the subtheme "*Peer pressure"*. I recommend revisiting the transcripts and coding to confirm this alignment.

Regarding Table 3, it is unclear how the recommendations for preventing overconsumption of alcohol and salt were developed. Were these recommendations explicitly derived from the Socio-Ecological Model framework, or were they based on the structure of the interview guide? This should be clarified in the Methods section so readers can understand the process used to establish these recommendations.

Discussion

The discussion provides a strong conceptual application of the Socio-Ecological Model (SEM) to frame the drivers of excessive salt and alcohol consumption in Ghana. The writing is generally coherent and well-structured, effectively utilising literature to support its arguments. However, several aspects could be strengthened for clarity, depth, and balance.

Page 9, lines 8-30: Paragraphs 1 to 3 seem to show the same thing regarding the SEM framework or model. The citations are numerous but occasionally repetitive, especially number 31, as it appears multiple times without clear differentiation, which interrupts the narrative flow.

Page 9, lines 46-47: "*A majority of Ghanaian alcohol consumers (57%) prefer…*". They are adults, young adults, youth, or the elderly? Consider specifying the age group if youth is a concern.

Page 10, lines 7-15: Here, the authors acknowledge a gap in understanding the interaction between salt and alcohol consumption, but don't offer any hypotheses or theoretical expectations. Propose specific pathways or mechanisms by which alcohol might influence salt consumption (e.g., appetite regulation, social settings) and suggest methodological approaches for future studies (e.g., mixed-methods, longitudinal).

Lastly, the discussion is lacking of critical comparison with other contexts or literature, while this is important for generalisation. It is because the discussion is very Ghana-centric, which is valuable for context, but it lacks comparison to other LMICs or global trends. It would be better to briefly compare findings with studies from other Sub-Saharan African countries or global literature to emphasise transferability or uniqueness.

Conclusion

Page 10, lines 30-38: While the conclusion notes that interventions should target all SEM levels, it lacks concrete examples or recommendations. Briefly mention specific implications (e.g., educational campaigns, licensing reforms, or community engagement strategies) that could emerge from the findings.

The conclusion misses an opportunity to acknowledge limitations or call for future research, as done in the discussion. Add one sentence to frame areas for further research, e.g., Future research should explore how multi-level interventions interact to produce sustained dietary changes in diverse cultural contexts.

VERSION 2 - AUTHOR RESPONSE

**Reviewer's Comment**	**Response from Authors**
**Reviewer #3: Prof. Jelena Jovicic-Bata, University of Novi Sad**	
**Abstract**<br>The abstract is informative enough and adequately summarizes the main findings.<br><br>**Strengths and limitations**<br>Well written.	We thank the reviewer for this positive assessment of the abstract and strengths and limitations. We are glad that the summary of our work was found to be clear and informative.
**Introduction**<br>Introduction is comprehensively written and uses current references.<br><br>In the first paragraph, lines 37-45, it would be advisable to add at least some information on the usual salt content of traditional cuisine, as	 <br><br><br>We thank the reviewer for this helpful suggestion. We have revised the introduction to acknowledge the contribution of traditional

this is also an important determinant of the overall salt intake. The way this paragraph is written at the moment implies that globalization and urbanization are the sole drivers of excessive salt intake.	Ghanaian dishes (e.g., soups, stews, and salted fish) to overall salt intake. (Lines 78-80)
**Materials and methods**<br>This section is written concisely and is well organized, provides all the necessary elements.	We thank the reviewer for this positive feedback.
**Results**<br>The results are easy to understand and follow, concise and comprehensive.<br><br>Page 6 if 20, lines 22-26, the Authors state that "Stakeholders also noted that alcohol is overconsumed for non-recreational purposes, including as an aphrodisiac or antidepressant. These beliefs persist despite limited evidence supporting such claims (27, 28)." Are these claims about alcohol being an aphrodisiac and/or antidepressant culturally based? If that is the case, that could be mentioned here, as in many other cultures, alcohol is not seen as such.	We thank the reviewer for this positive feedback and are pleased that the results section was found to be clear and comprehensive.<br><br>We thank the reviewer for this insightful comment. We agree that these beliefs are culturally based and have revised the text to clarify that the perceptions of alcohol as an aphrodisiac or antidepressant are rooted in cultural contexts in Ghana. (Line 240)
**Discussion**<br>Discussion is well-led, goes in line with the main findings and is comprehensive.<br><br>Page 10 of 20, lines 3-6: This might be the place to possibly elaborate in one paragraph on the underrepresentation of salt and alcohol industry representatives – What do the Authors think would be the main differences between their answers compared to other stakeholders included in the study?	We thank the reviewer for this thoughtful suggestion. We have expanded the discussion to acknowledge that including industry representatives could have introduced perspectives shaped by commercial interests, such as prioritising profit, which may differ from the public health-focused views of other stakeholders. This clarification strengthens the limitation statement. (Lines 418-422)
**Reviewer #4: Dr Andi Thahir, The University of Sydney, Hasanuddin University School of Public Health**	
**Introduction**<br>The flow of the introduction does not feel smooth, likely due to the way subheadings have been assigned to paragraphs. The authors should consider reorganising this section into three major areas: why salt and alcohol consumption matter in general, how prevalent and serious these issues are in Ghana, and what the current knowledge gaps are, along with potential solutions. Additionally, it remains unclear why salt and alcohol consumption in	We thank the reviewer for these helpful suggestions. We reorganised the introduction, removing the subheadings to make it more narrative and smoother, while strengthening the study rationale (Lines 64-73).<br><br>We also clarified why the Ghanaian context warrants urgent attention by highlighting early-onset hypertension, cultural embedding of

Ghana should be viewed as an urgent concern beyond the observation that both consumption rates are high.<br><br>There is no discussion of whether these two factors exacerbate each other's health impacts, whether they share mechanistic pathways, or why their combined burden warrants particular attention. Clarifying these points would strengthen the rationale and make the introduction more compelling.	alcohol, and the influence of the alcohol industry (Lines 78-109).<br><br>We also added a note that salt and alcohol may compound health impacts, particularly through blood pressure and cardiovascular risk (Lines 61-63).
**Method**<br>Page 4, lines 43-45: You mentioned, "The sampling frame included health policymakers, practitioners, and researchers from key institutions in Ghana. A total of 25 stakeholders were initially approached, of whom 21 agreed to participate (Table 1)." Could the authors clarify what prevented the inclusion of all 25 participants? How did you determine when saturation of responses had been reached? Additionally, how was initial contact with participants made, and who were considered the key informants? Was the interview conducted in English or local languages such as Akan or Ewe?<br><br>Page 5, lines 23-25: It is stated that "Additionally, initial results were presented to participants at a workshop in Ghana in May 2024, where feedback was incorporated before finalising conclusions." Could you clarify what kind of feedback was received and what the purpose of collecting this feedback was? Did the feedback lead to changes in the initial results, particularly if there were discrepancies between the interview data and participant reflections during dissemination?<br><br>It would also be helpful to explain more specifically how initial codes were developed inductively. For example, what criteria guided the identification of patterns before mapping them onto the SEM levels?<br><br>I agree with the previous round of review asking, "How was the interview guide developed, based on?" Although the authors claimed that they had already revised it but the response and revision did not actually and sufficiently address it. They mentioned, "A	We thank the reviewer for these queries. We clarified in the Methods that four participants declined due to scheduling conflicts, interviews were in English (Line 167), and saturation was reached when no new themes emerged. (Lines 145-148)<br><br>Additionally, we thank the reviewer for this question. The workshop feedback validated the identified themes and offered minor clarifications, without changing the core results. (Lines 192-194)<br><br>We have clarified in the Methods that initial codes were developed inductively based on recurring concepts, emphasis by participants, and relevance to drivers of salt and alcohol consumption. (Lines 186-187)<br><br>The interview guide was developed based on the study objectives and relevant literature on salt and alcohol consumption, and then refined through review and feedback from two experts in nutrition and public health, who are co-authors on this paper. (Lines 163-166)

nutrition expert contributed to refining the interview guide" How? Also, are these experts acknowledged in the acknowledgement or as co-authors?	
**Results**<br>Table 2 shows that "Delicacies rich in salt" is categorised under the "Sociocultural factors" theme. However, this appears to reflect participants' opinions and perceptions, which are more likely shaped by their intrapersonal attitudes. Therefore, it may fit better under the "Perceptions and Beliefs" theme. Does this subtheme overlap with "Prioritising taste"?<br><br>Meanwhile, the "Use of salt for food preservation" seems more appropriately placed under the Sociocultural factors theme. What is your view on this?<br><br>Importantly, I think "Household cooking and food preparation behaviour" should be classified under the Community SEM level within the "Sociocultural factors" theme. If this reclassification is made, then the Interpersonal domain cannot be represented solely by the subtheme "Peer pressure". I recommend revisiting the transcripts and coding to confirm this alignment.<br><br>Regarding Table 3, it is unclear how the recommendations for preventing overconsumption of alcohol and salt were developed. Were these recommendations explicitly derived from the Socio-Ecological Model framework, or were they based on the structure of the interview guide? This should be clarified in the Methods section so readers can understand the process used to establish these recommendations.	We thank the reviewer for this thoughtful observation. To clarify the sociocultural dimension, we revised the subtheme to "traditional dishes rich in salt," which better reflects cultural food practices rather than individual taste preferences. This distinguishes it from the intrapersonal theme of "prioritising taste." (Table 2)<br><br>We agree that salt use for preservation is culturally embedded and have retained it under the "Sociocultural factors" theme in the Community level, rather than as a broader societal factor. (Table 2)<br><br>After revisiting the transcripts, we retained "Household cooking and food preparation behaviour" under the Interpersonal level, as participants emphasised the influence of family members' cooking practices and shared meals within households. This reflects interpersonal dynamics more than broader community norms, which are captured separately under the Community–Sociocultural theme.<br><br>We clarify in the Methods that the recommendations in Table 3 were primarily derived from the structure of the interview guide, which was also designed to elicit participants' views on strategies for preventing overconsumption. (Lines 164-166)
**Discussion**<br>The discussion provides a strong conceptual application of the Socio-Ecological Model (SEM) to frame the drivers of excessive salt and alcohol consumption in Ghana. The writing is generally coherent and well-structured, effectively utilising literature to support its arguments. However, several aspects could be strengthened for clarity, depth, and balance.	

Page 9, lines 8-30: Paragraphs 1 to 3 seem to show the same thing regarding the SEM framework or model. The citations are numerous but occasionally repetitive, especially number 31, as it appears multiple times without clear differentiation, which interrupts the narrative flow.<br><br>Page 9, lines 46-47: "A majority of Ghanaian alcohol consumers (57%) prefer…". They are adults, young adults, youth, or the elderly? Consider specifying the age group if youth is a concern.<br><br>Page 10, lines 7-15: Here, the authors acknowledge a gap in understanding the interaction between salt and alcohol consumption, but don't offer any hypotheses or theoretical expectations. Propose specific pathways or mechanisms by which alcohol might influence salt consumption (e.g., appetite regulation, social settings) and suggest methodological<br>approaches for future studies (e.g., mixed-methods, longitudinal).<br><br>Lastly, the discussion is lacking of critical comparison with other contexts or literature, while this is important for generalisation. It is because the discussion is very Ghana-centric, which is valuable for context, but it lacks comparison to other LMICs or global trends. It would be better to briefly compare findings with studies from other Sub-Saharan African countries or global literature to emphasise transferability or uniqueness.	We thank the reviewer for this observation. We have streamlined the opening section of the Discussion by reducing repetition, and citing reference 31 appropriately to improve clarity and flow. (Lines 376-382)<br><br>We thank the reviewer for this helpful point. The statistic refers to adults, and we have clarified this in the text. (Line 406)<br><br>We have revised the Discussion to propose possible pathways and suggested future research using mixed-methods and longitudinal designs to explore these interactions. (Lines 427-431)<br><br>We thank the reviewer for this important suggestion. We have revised the Discussion to situate our findings within the broader literature, noting similarities with other sub-Saharan African contexts and highlighting how the identified drivers may also be relevant in global settings. This addition emphasises both the transferability and the contextual uniqueness of the results. (Lines 432-437)
**Conclusion**<br>Page 10, lines 30-38: While the conclusion notes that interventions should target all SEM levels, it lacks concrete examples or recommendations. Briefly mention specific implications (e.g., educational campaigns, licensing reforms, or community engagement strategies) that could emerge from the findings.<br><br>The conclusion misses an opportunity to acknowledge limitations or call for future research, as done in the discussion. Add one sentence to frame areas for further research, e.g., Future research should explore how multi-	We thank the reviewer for this helpful suggestion. We have revised the Conclusion to include specific examples of interventions that align with the identified SEM levels. (Lines 459-463)<br><br>We have also added a sentence to the Conclusion calling for future research on how multi-level interventions interact to influence

level interventions interact to produce sustained dietary changes in diverse cultural contexts.	dietary behaviours across different cultural contexts. (Lines 465-467)

VERSION 3 - REVIEW

**Reviewer**	**3**
**Name**	**Jovicic-Bata, Jelena**
**Affiliation**	**University of Novi Sad, Department of Pharmacy**
**Date**	**22-Sep-2025**
**COI**	

I thank the authors for addressing all my concerns and raised issues in a constructive and practical manner.

Just for clarification, I believe that the names of us, reviewers, have been mixed up in Authors' response letter as I can see my previous comments under someone else's name.
